# *N*-Glycosylation Profile of Abrin Certified EU Reference Material

**DOI:** 10.3390/toxins17030108

**Published:** 2025-02-26

**Authors:** Roland Josuran, Andreas Wenger, Sylvia Worbs, Bettina Kampa, Andreas Rummel, Brigitte G. Dorner, Sabina Gerber

**Affiliations:** 1 Institute of Chemistry and Biotechnology, ZHAW Zurich University of Applied Sciences, 8820 Wädenswil, Switzerland; 2 Biological Toxins (ZBS3), Centre for Biological Threats and Special Pathogens, Robert Koch Institute, 13353 Berlin, Germanydornerb@rki.de (B.G.D.); 3 Institute of Toxicology, Hannover Medical School, 30625 Hannover, Germany; rummel.andreas@mh-hannover.de

**Keywords:** abrin, *N*-glycosylation, glycan profile, abrin isoforms

## Abstract

Abrin is a highly toxic plant protein encompassing four isoforms, abrin-a, -b, -c and -d. An abrin reference material was isolated from *Abrus precatorius* and certified (EURM-113) by the EuroBioTox consortium. Here, we present a detailed characterisation of the *N*-glycosylation profile of EURM-113. The monosaccharide composition of the *N*-glycans was determined and quantified. Release of the *N*-glycans yielded 13 different partially xylosylated, oligomannosidic and paucimannosidic glycan structures. Two *N*-glycans were found at N82 and N110 of the abrin-b A-chain and another two at N100 and N140 of the B-chains. The *N*-glycosylation sites N200 in the A-chain and N141 in the B-chain were non-glycosylated. Whereas N82 and N110 of abrin-b comprised paucimannosidic glycans, N100 and N140 of the B-chains revealed oligomannosidic *N*-glycans. Xylose was absent in the glycans at N100 but was present in about half of the glycans at N140. Hence, this study revealed substantially different types of glycan structures within the B-chains compared to the abrin-b A-chain. Furthermore, the most C-terminal *N*-glycosylation site in the A-chain was found to be non-glycosylated in all abrin isoforms detected. Additionally, the establishment of the *N*-glycosylation profile of the abrin reference material led to the identification of the abrin isoforms -a, -b and -c. In conclusion, the abrin *N*-glycosylation profile is highly similar to the one of ricin and yields high analytical value to be further exploited as a fingerprint in forensic investigations to uncover toxin production or toxin provenance.

## 1. Introduction

Abrin is a highly potent plant toxin [1] and belongs to the type II ribosome-inactivating proteins (RIPs II) consisting of an A-chain comprising the catalytic *N*-glycosidase activity, which inactivates the 60s ribosomal subunit, and a lectin-like B-chain, which binds D-galactose [2]. It is located in the vacuoles of the seeds of *Abrus precatorius*, where it serves as protection against predators and fungi due to its high toxicity [3].

More than 30 years ago, the sequences of the four isoforms abrin-a, -b, -c and -d were obtained from the mRNA of the seeds [4] and from the genome of the leaves [5], and they were reported with high amino acid sequence identity of above 80% for the A-chains and higher than 93% for the B-chains. In 2018, a whole genome shotgun sequence was deposited from the transcriptome and the genome of the leaves of *Abrus precatorius*, revealing abrin-like transcripts sharing 70.0–77.1% amino acid sequence identity with abrin-a, -b and -d and 94.5% and 97.7% amino acid sequence identity with the abrin-c isoform previously deposited [6] (Supplementary Figure S1). The homology of the isoforms abrin-a, -b, -c and -d is also reflected by their similar lethality of 10, 25, 16 and 31 µg/kg body weight, respectively [7].

The B-chain of all abrin isoforms contains three conserved *N*-glycosylation motifs at N100, N140 and N141. N100 and one of the two other asparagine residues (N140 or N141) were reported to be glycosylated with M6 or M7 and M4X, M5 or M6, respectively, whereas Mn stands for n number of mannose moieties attached to the conserved core chitobiose and X indicates one xylose [8,9] (glycan nomenclature as described previously [10]). Within the A-chain, only the *N*-glycosylation motif at position N200 is strictly conserved in all abrin isoforms. Abrin-b contains two additional motifs at N82 and N110 [4]. However, *N*-glycosylation at any of the three motifs of the A-chains has not been reported to date.

Abrin was the agent in several poisoning incidents [11], assassination attempts [12,13] and biothreat scenarios [14,15,16], and hence, it is listed as a dual-use item according to Annex 1 of regulation EU 2021/821 in Europe [17] and as one of the US HHS Select Agents and Toxins [18]. Abrin is one of the nine most likely relevant biotoxins applying the General Purpose Criterion of the Chemical Weapons Convention, which is monitored by the Organisation of the Prohibition of Chemical Weapons (OPCW) following the recent report and the recommendations of the Scientific Advisory Board’s Temporary Working Group on the Analysis of Biotoxins [19].

In this context, the European Commission funded the EuroBioTox project, which produced and subsequently certified an abrin EU reference material (EURM-113) [10,20,21,22]. As part of the in-depth characterisation of EURM-113, its *N*-glycosylation profile is described in the present study.

## 2. Results

The elucidation of the glycan profile of abrin encompassed the determination of the monosaccharide composition of the glycans by ion chromatography as a first step. Further, the released glycans were fractionated according to their monosaccharide composition, followed by separation of the isomeric glycans, with identification by mass spectrometry.

### 2.1. Monosaccharide Analysis

**Figure 1:**
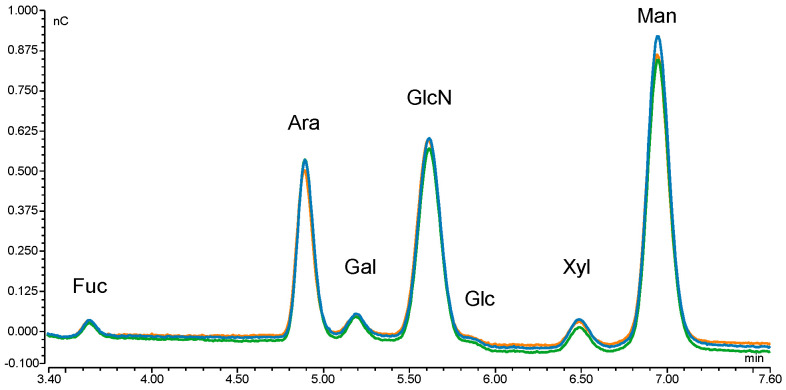
Monosaccharide determination of abrin EURM-113 using HPAEC-PAD. Overlay of the chromatograms of the triplicate analysis (one operator, parallel sample preparation) shown as green, blue and orange traces. Fucose (Fuc), arabinose (Ara), galactose (Gal), glucosamine (GlcN, converted from N-acetylglucosamine during acid hydrolysis), glucose (Glc), xylose (Xyl) and mannose (Man).

The monosaccharide composition of abrin was determined by total acidic hydrolysis of the carbohydrates and the monosaccharides were analysed using high pH anion exchange chromatography with pulsed amperometric detection (HPAEC-PAD), with arabinose added as the internal standard for quantification (Figure 1). Glucosamine (GlcN, formed from *N*-acetylglucosamine (GlcNAc) during hydrolysis), fucose (Fuc), xylose (Xyl) and mannose (Man) were detected, which represent monosaccharides expected from known plant *N*-glycans. Galactose (Gal) and glucose (Glc) were additionally identified, which are not reported to be part of the *N*-glycans of vacuole plant proteins [23]. Galactose most likely resulted as an impurity from the purification process (since a galactose-containing buffer was used for a chromatographic elution step), whereas glucose was likely introduced into the sample as trace impurities from paper wipes [10,24].

**Figure 2:**
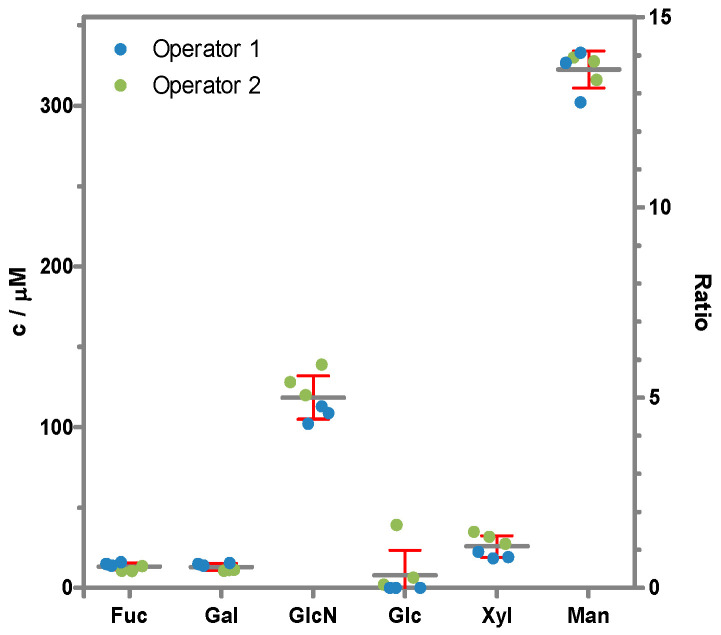
Monosaccharide content of abrin. Absolute concentration of monosaccharides (left axis) determined by HPAEC-PAD of the hydrolysed samples by two operators (green and blue dots) performing three sample preparations each. The mean values and standard deviations are shown by grey lines and red whiskers, respectively. The monosaccharide to protein ratio was calculated based on the certified protein concentration of EURM-113 (right axis).

The absolute concentrations of the released monosaccharides and their proportions per mole of protein were determined using HPAEC-PAD and are represented in Figure 2. The acidic hydrolysis was performed by two different persons on different days in triplicate each time. The highest proportions were obtained for Man and GlcN (originating from GlcNAc) with 318 µM and 117 µM, respectively, and lower concentrations of Fuc and Xyl (13 µM and 25 µM, respectively), which led to the assumption that abrin was mainly glycosylated with oligomannosidic glycans and to a minor share with paucimannosidic glycans (Table 1). The molar ratios of GlcN and Man to protein determined were 5.0 and 13.6, respectively. Since oligo- and paucimannosidic *N*-glycans contain the strictly conserved chitobiose core comprising two GlcNAc residues [23], a mean stoichiometry of 2.5 glycans resulted per abrin molecule, with an average content of 5.4 Man moieties per glycan.

### 2.2. Identity and Relative Content of Released Glycans

**Figure 3:**
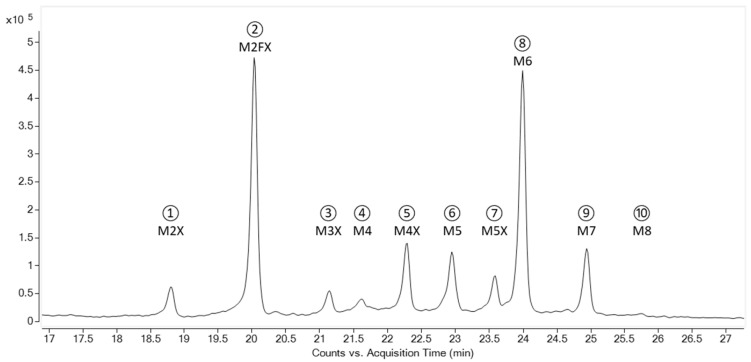
Separation of the glycans of abrin according to the monosaccharide composition. Released glycans were chromatographed and detected with HILIC-MS. M (mannose) with its number attached to the core chitobiose, X (xylose), and F (fucose). The numbered peaks were fractionated and used for further analyses.

*N*-glycans were released by hydrazinolysis, followed by an *N*-reacetylation of GlcN and reduction of the reducing end, resulting in intact alditol glycans. The glycans were separated using hydrophilic interaction liquid chromatography mass spectrometry (HILIC-MS) to detect the glycans according to their number of monosaccharides. The HILIC-MS chromatogram of the extracted m/z of the singly protonated glycan ions yielded ten glycan peaks with monosaccharide compositions as depicted in the peak labels (Figure 3).

**Figure 4:**
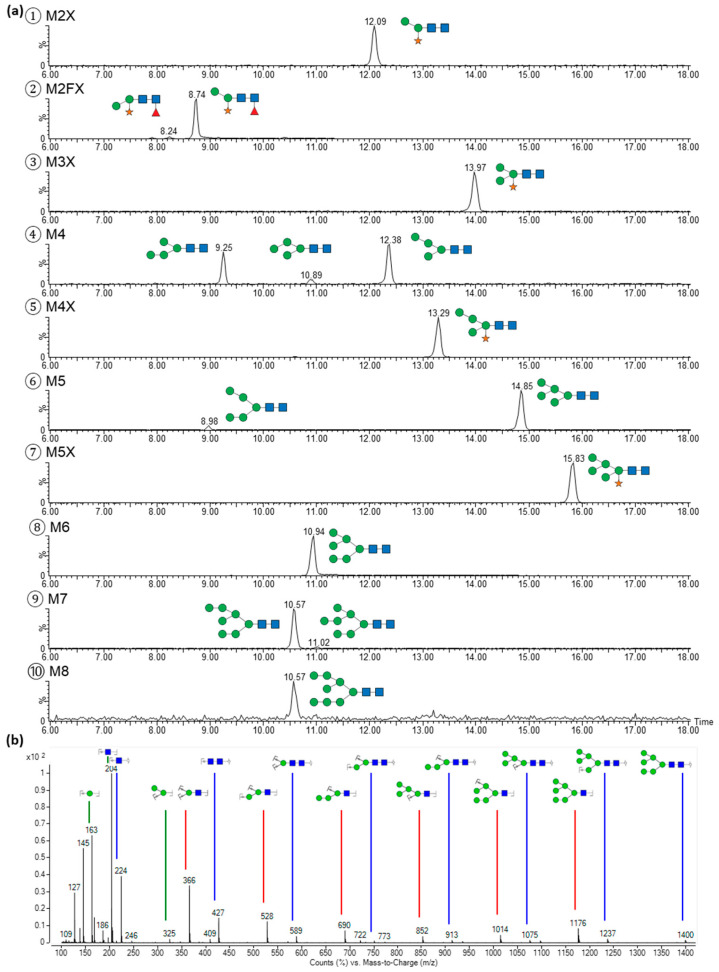
Identification of the glycans from abrin in the HILIC peaks. (**a**) Glycans in the fractions from the HILIC were separated according to the structures using PGCC-MS. Traces of singly deprotonated glycan ions were extracted. (**b**) Glycans were fragmented using collision-induced dissociation to elucidate the monosaccharide sequence. A representative spectrum of M6 is shown. Green circle (mannose), orange star (xylose), red triangle (fucose), and blue square (*N*-acetylglucosamine).

The glycans were fractionated and re-chromatographed by HILIC-MS to confirm the absence of cross-contaminations with other glycans and the glycans were further analysed orthogonally by porous graphitised carbon chromatography (PGCC)-MS in order to separate potential isomeric species of an identical monosaccharide number contained in the HILIC peaks [25]. The purity of the glycans in all the fractions was determined to be >95% (Figure 4), a prerequisite for elucidation of the glycan structures with specific glycosidases.

Figure 4 shows the PGCC-MS analysis of the glycan fractions from the HILIC. The retention times of the glycans were compared with commercial and self-prepared references and the monosaccharide sequences were determined by tandem MS analysis. The fractions from the HILIC were additionally subjected to hydrolysis with different specific exoglycosidases. The products were evaluated by PGCC-MS and their formation rates were employed to assign the linkage configuration of each glycan. Hence, identification of the released *N*-glycans was established by a combination of the molecule and fragment masses, the retention time in HILIC and PGCC compared to the reference glycans and the use of specific exoglycosidases, similarly to the approach suggested by Pabst et al. [26]. For the glycans M2X, M3X, M4X, M5X, M6 and M8, only one linkage configuration was detected each. Paucimannose M2FX (F stands for α(1-3)-linked core fucose, refer to Table 2 for the structures) and oligomannosidic glycans with five and seven mannose moieties resulted in two elution peaks in the PGCC each. The isomeric species M2FX (2) and M2FX (1), M5 (1,3) and M5 (1,2), as well as M7 (1) and M7 (2) differ in terms of the antennary locus of the mannose residues at the non-reducing end (numbers in parenthesis describe the linked antenna). The M4 fraction was separated in three different structures with M4 (3), M4 (2) and M4 (1). Hence, a total of 15 different glycan structures were identified in abrin EURM-113.

**Figure 5:**
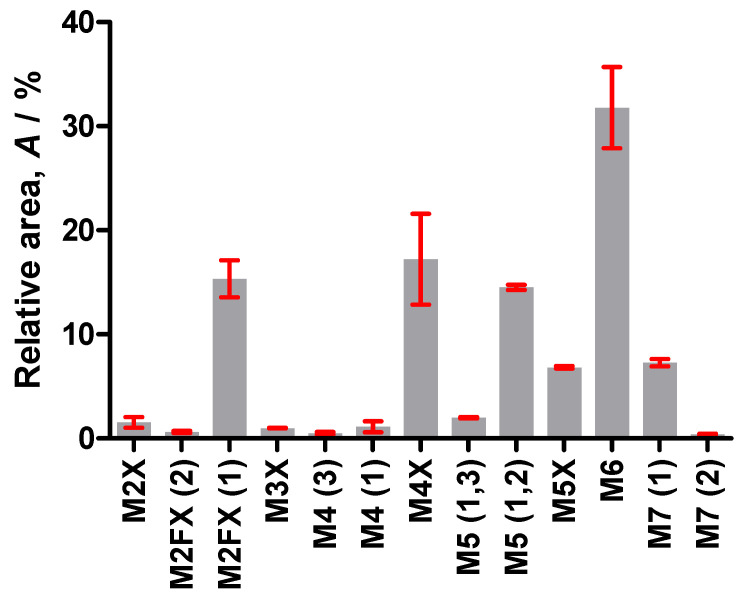
Relative quantities of the glycan structures of abrin. The released glycans were analysed by PGCC-MS and the shares were calculated based on the peak areas in the extracted ion chromatograms of the singly and doubly charged ions. Mannose moieties (M) with the number, xylose (X), fucose (F); numbers in parentheses according to the antennary locus of the mannose moieties [10], core chitobiose is implicit. Error bars represent the standard deviation of the replicate analyses from two operators on different days in triplicate from individual vials.

The relative amounts of the different glycan structures were determined. For this, *N*-glycans were released from the protein backbone of abrin by two different operators on two different days in three replicates from three different vials, each using hydrazinolysis. The released glycans were analysed by PGCC-MS in positive mode, which is best suited for quantitative measurements of glycans [26]. The relative quantities were calculated from the peak areas in the extracted ion chromatograms of the singly and doubly charged ions (Figure 5 and Table 2).

M4 (2) and M8 were not detected in the analysis applied for quantification. M4 (2) coeluted with M6 in the PGCC-MS, hampering discrimination between M4 (2) and the in-source fragment M4 originating from M6. PGCC-MS analysis of the HILIC fractions showed that M4 (2) accounted for approximately 6% of the total amount of M4. Also, M8 revealed very low intensity; thus, it was most likely present below the lower limit of quantification in this analysis. As the shares of these glycans were very low, the effect on the proportions of the other glycans was not significant.

More than 80% of the glycans constituted oligomannose with or without xylose, reflecting the high share of mannose, which was determined by monosaccharide analysis. Xylosylated oligo- and paucimannosidic glycans accounted for 42% and the two fucosylated paucimannoses M2FX (2) and M2FX (1) for 16%. This finding was in agreement with the lower share of xylose and fucose as identified in the monosaccharide analysis.

### 2.3. Glycopeptide Analysis

**Figure 6:**
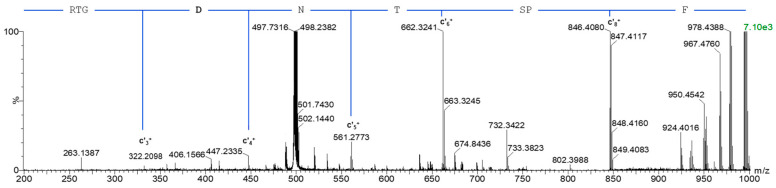
Glycosylation of the B-chain N140 of abrin. Fragment spectrum of the chymotryptic peptide ^137^RTGNNTSPF_145_ after PNGase F treatment acquired with ETD confirms aspartate and, hence, glycosylation at N140.

Two *N*-glycosylation motifs at N140 and N141 in the B-chain are in direct neighbourhood. Chymotryptic digestion of abrin yielded the peptide ^137^RTGNNTSPF_145_ and the following deglycosylation using PNGase F converted asparagine to aspartate, which revealed the locus of *N*-glycans exclusively at N140 and excluded *N*-glycosylation at N141 when applying RPLC-MS with electron transfer dissociation (ETD). Figure 6 shows the fragment ion spectrum of this peptide with the detection of the singly charged c’ ions 3, 4, 5, 6 and 8, whereas c’_7_ was not formed due to the proline at position 144. The difference in c’_3_ and c’_4_ matched the mass of aspartate and the difference in c’_4_ and c’_5_ corresponded to the mass of asparagine, which confirmed the original glycosylation at N140 and corroborated the non-modified N141.

**Figure 7:**
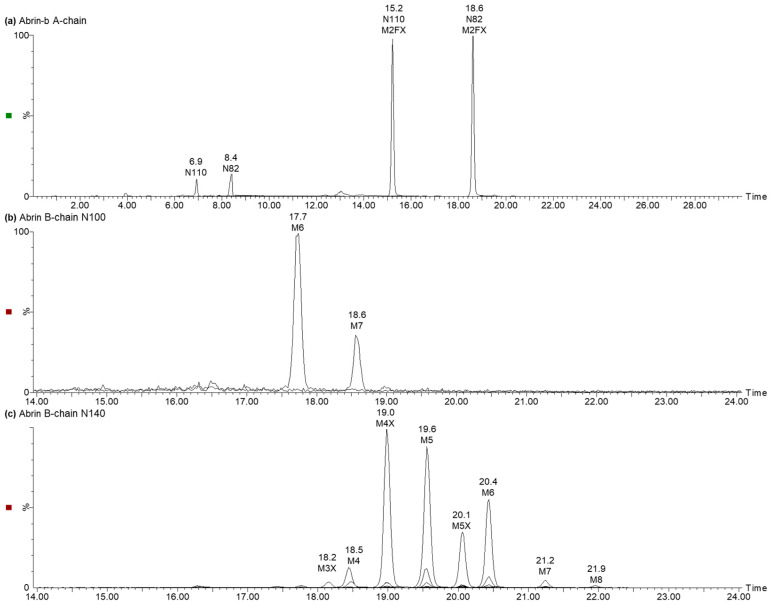
Glycopeptides of the abrin A- and B-chain showing the occupancy and glycan distribution. HILIC-MS chromatograms of the (**a**) tryptic peptides ^80^AGNR_83_ covering N82 and ^109^FNGSYIDLER_118_ covering N110, the two glycosylation motifs unique for the abrin-b A-chain, (**b**) chymotryptic peptide ^99^DNGTIINPKSALVL_112_ with N100 and (**c**) chymotryptic peptide ^137^RTGNNTSPF_145_ with N140 of the B-chain, with the glycosylation motifs conserved in all isoforms. Mannose moieties with the numbers indicated (M), xylose (X), and fucose (F). Small signals with similar retention times as the glycopeptide peaks detected originated from the in-source decay of larger glycans and, thus, represented artefacts.

The chymotryptic peptides ^99^DNGTIINPKSALVL_112_ and ^137^RTGNNTSPF_145_ of the B-chains were conserved in all abrin isoforms and were used for analysis of the *N*-glycans at N100 and N140, respectively. The type and stoichiometry of the *N*-glycans attached to N82 and N110 of the abrin-b A-chain were determined with the unique tryptic peptides ^80^AGNR_83_ and ^109^FNGSYIDLER_118_, respectively. The peptides were identified using HILIC-MS and the peak areas of specific ions were used to calculate the shares of the glycans per glycosylation site. Non-glycosylated peptides of the abrin-b A-chain covering N82 and N110 were detected at 8.4 min and 6.9 min, respectively, and M2FX glycopeptides at 18.6 min and 15.2 min for N82 and N110, respectively (Figure 7a). The occupancy amounted to 92% and 94% for N82 and N110, respectively. The peptides covering the strictly conserved *N*-glycosylation site N200 of the A-chain are unique for each of the four isoforms (Supplementary Figure S2). However, only the non-glycosylated forms of these peptides were identified for abrin-a, -b and -c, whereas neither the glycosylated nor non-glycosylated peptide of abrin-d was detected by RPLC-MS.

**Figure 8:**
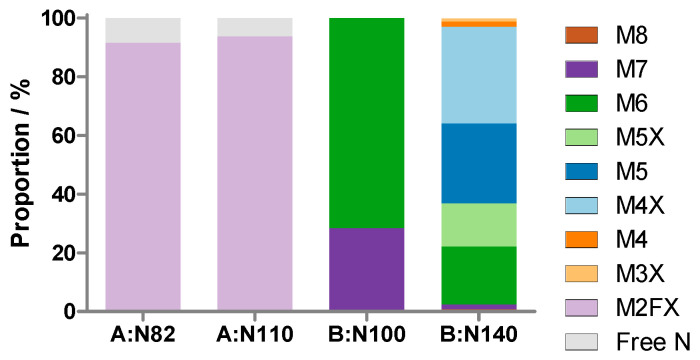
Proportions of the glycans detected at four different N-glycosylation sites of the abrin A-chain (A:N82, A:N110) and B-chain (B:N100, B:N140) of the glycopeptides. The two A-chain glycosylation sites A:N82 and A:N110 are unique to abrin-b. The two B-chain glycosylation sites B:N100 and B:N140 are conserved among all four abrin isoforms, -a, -b, -c, and -d. The glycosylation motifs N200 of the A-chain and N141 of the B-chain conserved in all four isoforms are non-glycosylated and are therefore not represented in the figure.

Non-glycosylated peptides were not detected for the two peptides covering N100 and N140. The glycopeptide covering N100 of the B-chain occupied with either M6 or M7 eluted at 17.7 min and 18.8 min, respectively (Figure 7b). N140 of the B-chain was heterogeneously glycosylated with oligomannosidic glycans with or without xylose (Figure 7c). The main forms found were M4X, M5, M5X and M6. The shares of the different *N*-glycans per glycosylation site are summarised in Figure 8.

### 2.4. Characterisation of Intact A- and B-Chains

**Figure 9:**
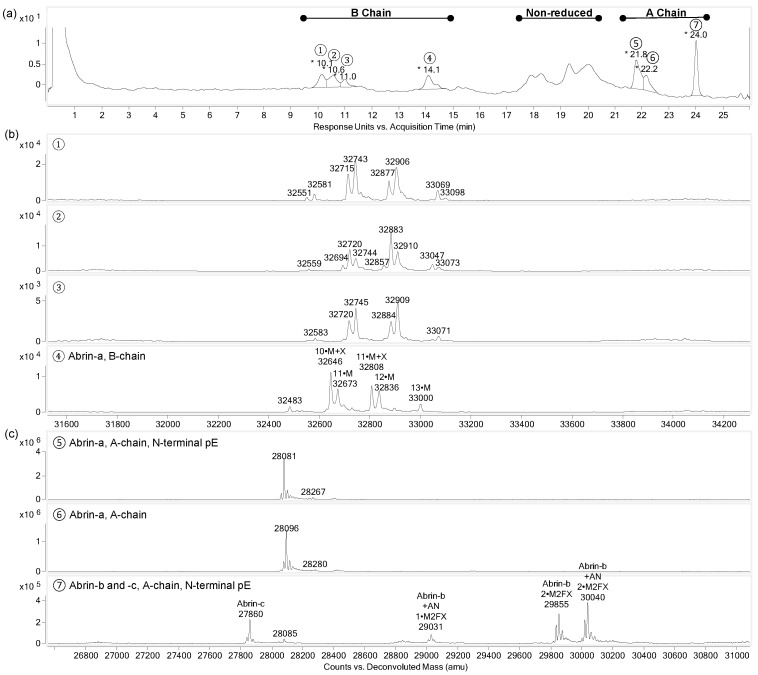
RPLC-MS analysis of the intact A- and B-chains of abrin. (**a**) RPLC chromatogram (UV trace, λ = 214 nm) of reduced abrin. B-chains peaks 1–4; A-chains peaks 5–7. The peaks between 16 and 21 min were non-reduced abrin species resulting from incomplete reduction. (**b**) Deconvoluted mass spectra of chromatographic B-chain peaks 1–4. M and X stand for mannose and xylose, with the total number of mannose residues on the chitobiose core at two glycosylation sites. (**c**) Deconvoluted mass spectra of chromatographic A-chain peaks 5–7. The N-terminal glutamine converted to pyroglutamate is indicated with pE. The peaks are labelled with the number of M2FX glycosylation and +AN when an additional dipeptide remained at the C-terminus from the linker peptide.

To corroborate the assignment of the glycans to the peptides and, hence, to the two polypeptide chains, abrin was reduced and intact chains were analysed by RPLC-MS (Figure 9). Peaks 5 and 6 show the masses corresponding to the abrin-a A-chain with glutamine and the respective metabolised pyroglutamic acid at the N-terminus (Figure 9a,c). In agreement with the glycopeptide data, the A-chain of abrin-a found was devoid of any *N*-glycan. Peak 7 represents the A-chain of abrin-b and abrin-c. The abrin-b A-chain featured N-terminal pyroglutamic acid and—in agreement with the data from the peptide analysis—was modified with two M2FX glycans or, to a minor share, with a single M2FX glycan (Figure 9a,c). The C-terminal amino acid of the A-chain of abrin-b was N250, confirming the results of Hung et al. [4], but also another C-terminus with two additional amino acids (^251^AN_252_) from the linker peptide was identified and confirmed by peptide analysis. The A-chain of abrin-c was detected with N-terminal pyroglutamic acid but without any glycan modification, which correlated with the glycopeptide results (Figure 9c).

Whereas the masses of the A-chains matched well with the calculated masses from the amino acid sequences (Supplementary Table S1), the B-chains could not be assigned unambiguously. The mass spectra of chromatographic peaks 1 to 4 showed mass peaks with differences of 162 and 132 Da, corresponding to mannose and xylose, respectively. Since the A-chains were either non-glycosylated or homogenously glycosylated with M2FX, whereas the B-chains showed nearly full occupancy with multiple different oligomannosidic glycans, in agreement with the glycopeptide data, chromatographic peaks 1 to 4 were assigned to the B-chain. This assignment was further supported by the generally higher molecular masses of the B-chains in the mass range of approximately 32.4 kDa to 33.2 kDa compared to the A-chains constituting approximately 27.8 kDa to 30.2 kDa. The main mass of peak 4 matched the mass of the abrin-a B-chain with M6 at N100 and M4X at N140, which were the most abundant structures at these glycosylation sites also found for the glycopeptides. The other peaks in the mass spectra were varying combinations of oligomannosidic glycans with and without xylose. The peaks 1–3 in the chromatogram showed four prominent mass peaks with mass differences of mannose or xylose each. However, these masses could not be assigned to any of the abrin polypeptide chains, but identification as A-chains could be excluded due their inherently divergent mass range and due to their higher homogeneity of glycosylation, as have been elucidated from glycopeptides. Therefore, we concluded, that peaks 1–3 were presumably amino acid sequence variants of the B-chain abrin isoforms.

## 3. Discussion

**Figure 10:**
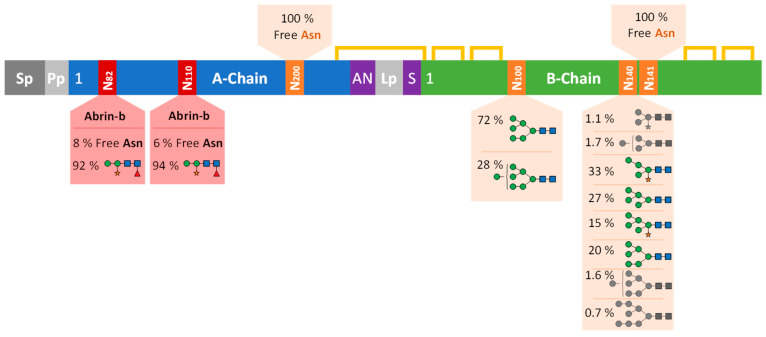
Schematic summary of the *N*-glycosylation profile of the abrin certified EU reference material EURM-113. The preproabrin with signal peptide (Sp), propeptide (Pp) and linker peptide (Lp) are shown in different shades of grey, while the A- and B-chains are represented in blue and green, respectively. The red boxes show the *N*-glycosylation sites unique to the abrin-b A-chain with the glycans identified and relative proportions thereof, while the orange boxes illustrate the *N*-glycosylation sites found in all the isoforms with glycans of a mixture of the isoforms of abrin-a, -b and -c detected in the sample. Yellow lines indicate disulphide bonds and purple boxes show amino acid variations in the single letter code of the respective polypeptide termini identified. The glycans detected at each site are depicted and coloured according to the Symbol Nomenclature for Glycans (SNFG), while structures with shares below 2% are shown in grey.

**Figure 11:**
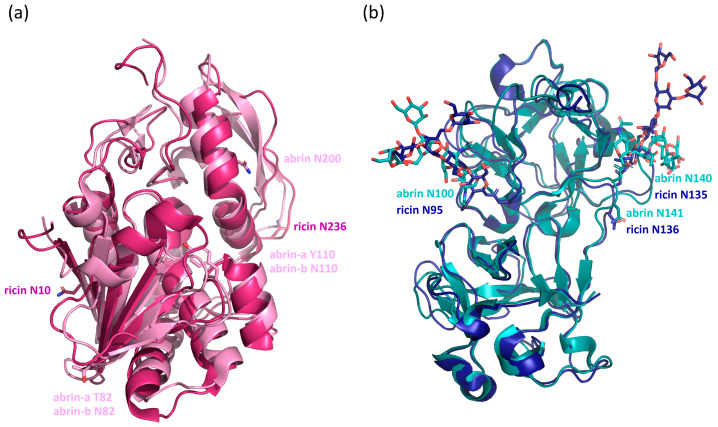
Superposition of the crystal structures of the A-chains (**a**) of abrin (bright pink) and ricin D (dark pink) and of the B-chains (**b**) of abrin (cyan) and of ricin D (blue). Protein backbones are illustrated as ribbons and glycan moieties as sticks. Asparagine residues located within *N*-glycosylation motifs are numbered and represented as sticks, revealing non-conserved loci of the *N*-glycosylation sites in the A-chains, but very high structural conservation in the B-chains. Additional loci of the *N*-glycosylation sites N82 and N110 unique for the abrin-b A-chain are depicted as sticks. The two consecutive asparagine residues within the B-chains (abrin N140, N141; ricin D N135, N136) encompass two consecutive *N*-glycosylation motifs in abrin, but only one motif (N135) in ricin D, with exclusive glycosylation of N140 of abrin resulting in strict structural conservation of glycan modification. Figure generated from structural data of natively sourced full-length abrin, PDB 1abr, and natively sourced full-length ricin D, PDB 2aai. Illustration produced in the PyMOL Molecular Graphics System, Version 3.0 Schrödinger, LLC.

In this study, the *N*-glycosylation profile, including the identification of the abrin isoforms, of the certified EU reference material (EURM-113) was established with the results summarised in Figure 10. The isoforms abrin-a [27], -b [28], and -c [29] were unambiguously identified by peptide data, which was further confirmed by masses of the intact polypeptide chains. In contrast, the tryptic peptides unique to abrin-d among the four isoforms were not detected ([30], Supplementary Figure S2). The data elucidated occupancy of >90% of M2FX glycans at N82 and N110 of the abrin-b A-chain, with the remarkable finding of the *N*-glycosylation motif at N200 devoid of any carbohydrate modification in abrin-a, -b, and -c. Hence, based on our data, the A-chains of abrin-a and abrin-c do not feature any *N*-glycosylation. The only published crystal structure of abrin presumably showing abrin-a solved from natively sourced material (PDB 1abr) did not resolve any glycans within the A-chain, but both B-chain *N*-glycosylation loci showed electron density for the core glycans (Figure 11) [9]. Our data show that the most N-terminal glycosylation site N100 in all B-chains was almost exclusively modified with M6 and M7 glycans of nearly 100% site occupancy, fully consistent with the proportions of the glycan structures and the occupancies reported in the literature [6] and in surprisingly high agreement with our data on the functionally and structurally closely related ricin [8]. Furthermore, we elucidated that only N140, and not N141 of the two consecutive *N*-glycosylation motifs of the B-chain was glycosylated, supporting the finding that NXT sites generally show higher rates of occupancy than NXS sites [31]. Moreover, these data are further reinforced by the high structural conservation of the *N*-glycosylation of abrin N140 and ricin N135 upon superposition of the crystal structures of natively sourced abrin (PDB 1abr) and natively sourced ricin D (PDB 2aai), revealing that abrin N141 devoid of any *N*-glycans superimposes on ricin N136, which lacks a glycosylation motif (Figure 11). N140 of the abrin B-chains showed heterogenous oligomannosidic glycosylation, either with or without xylose, similar to ricin but with a slightly reduced number of mannose residues and higher content of xylosylated forms [10]. The three most abundant glycans at N140 of abrin (M4X, M5 and M6) have also been described by Kimura [8] with slightly different proportions. Furthermore, our data revealed additional less abundant glycans at this glycosylation site. The abrin B-chain glycans could not be attributed to the different isoforms since the amino acid sequence of the glycopeptides was identical and the isoforms of the abrin material were not separated prior to the establishment of the glycosylation profile.

Overall, the data obtained for abrin revealed an *N*-glycosylation profile very similar to that of ricin [10]. The A-chain featured substantively higher glycan homogeneity with mainly paucimannosidic glycans in both toxins compared to the B-chain with heterogenous oligomannosidic glycans with partial xylose modification. Furthermore, the *N*-glycosylation sites close to the *N*-terminus of the A-chains of abrin-b and ricin revealed high occupancy in both RIP toxins, whereas the *N*-glycosylation motif close to the C-terminus of the A-chain showed very low occupancy or was free of any glycan modifications [10,32,33,34]. Higher occupancy is commonly observed at the N-terminally located *N*-glycosylation sites of polypeptide chains compared to the C-terminal ones [31].

The masses of the intact A-chain of all abrin isoforms and the B-chain of abrin-a were identified in high agreement with the calculated masses based on the published amino acid sequences (Supplementary Table S1). Additional polypeptide chains with the characteristic glycosylation pattern of the B-chains were detected but did not match the calculated mass of any of the published B-chain sequences. Two different primary structures of abrin-b have been published, one determined by cDNA sequencing [4] and one solved by analysis of the peptides [35]. Hence, the published amino acid sequence data may lack further sequence variations for the abrin B-chain. Moreover, our recently published study of the *N*-glycosylation profile of the closely related plant RIP II toxin ricin elucidated multiple sequence variants [10]. For these reasons, the primary structures of the B-chains of abrin-b, -c and maybe -d presumably inhere variations at single amino acid positions, which are not reported among the currently available sequences.

The definition of the precise glycosylation profile of abrin found in an investigation could be of forensic value to determine sample provenance or to identify corresponding sample batches. Different cultivars or origins of *Abrus precatorius* might reveal characteristic occupancies, structures and shares of *N*-glycans. In particular, the *N*-glycosylation site N200 free of any *N*-glycan modification in EURM-113 might feature *N*-glycosylation in other cultivars. Furthermore, the source of the material may exhibit variable shares of isoforms, which would be identified by the additional *N*-glycosylation sites N82 and N110 within the abrin-b A-chain. Finally, the protein *N*-glycosylation profile is generally highly unique for different organisms and is therefore a specific probe for the origin of its biosynthesis. Potential recombinantly produced material would not be discerned by its amino acid sequence and composition but would be distinguished primarily by a different overall *N*-glycosylation profile. Superposition of abrin and ricin crystal structures with very high structural similarity (rmsd 1.156 Å, 2835 atoms) further revealed that despite the highly similar glycosylation occupancy with very similar glycan structures in both A- and B-chains, glycosylation sites are located within non-conserved primary sequence regions and within different structural loci in the A-chains of both plant toxins, whereas the glycosylation sites within the B-chains show high sequence and structural conservation (Figure 11).

Multiple studies of the recombinantly produced A-chains of ricin and abrin-a, -b and -d in *Escherichia coli,* a host devoid of any endogenous *N*-glycosylation machinery, reported very similar in vitro ribosome-inhibiting activities compared to the A-chains of natively sourced toxins [36,37,38]. However, the ricin B-chain exhibited complete loss of asialofetuin binding upon mutagenesis of both *N*-glycosylation sites and expression in *Xenopus* oocytes [39]. Additional studies were performed by the same group, investigating ricin B-chain recombinantly expressed in *E. coli* and in tunicamycin-treated *Xenopus* oocytes [40], with controversial data obtained due to protein instability, as discussed by the group [39]. Concluding, the structurally conserved *N*-glycosylation sites with high overall occupancy within the B-chains of both plant toxins abrin and ricin presumably represent a prerequisite for either protein folding and solubility, or lectin functionality or both, whereas *N*-glycosylation within the A-chains of abrin and ricin, sites which are structurally non-conserved and reveal variable occupancy, allegedly is no requirement for protein integrity and in vitro enzymatic activity.

## 4. Materials and Methods

The certified abrin reference material EURM-113 was purified from *Abrus precatorius* at RKI and will be described in more detail in this Special Issue of Toxins (Worbs et al., manuscript in preparation). As abrin is a highly toxic substance, the intact material was handled exclusively in dedicated toxin facilities with restricted and controlled access under appropriate biosafety and biosecurity measures. The procedure for isolation was very similar to that published in [3,10,41]. The methods applied for abrin analysis are briefly described in the following part.

### 4.1. Qualitative and Quantitative Monosaccharide Analysis

Monosaccharides were released by acidic hydrolysis and analysed by HPAEC-PAD similarly as described by Hardy et al. [42]. The abrin EURM-113 samples were precipitated with 10% trichloroacetic acid and washed twice with acetone. The pellet was resuspended in 5 M trifluoracetic acid with 10 nmol/mL arabinose as the internal standard and hydrolysed at 100 °C for 3 h. The hydrolysates were dried using a SpeedVac (Thermo Fisher Scientific Inc., Waltham, MA, USA), resuspended in 10 µL 2-propanol, dried again and dissolved in water. The samples were diluted 100 times with water and analysed using HPAEC-PAD. The chromatography was performed on a Dionex ICS-5000+ ion chromatography system from Thermo Fisher Scientific Inc. with an electrochemical detector and equipped with a Dionex CarboPac SA10-4 μm (2 × 50 mm precolumn and 2 × 250 mm separation column Thermo Fisher Scientific Inc.). An isocratic elution was applied for 5 min at 3 mM potassium hydroxide, followed by a gradient to 100 mM potassium hydroxide within 0.5 min and a 2.5 min isocratic segment holding the concentration for column regeneration. Pulsed amperometric detection was performed with a gold working electrode and an Ag/AgCl pH reference electrode using the quadruple potential waveform.

### 4.2. Analysis of Released Glycans

HILIC-MS analysis of the released glycans with peak fractionation was performed on an Agilent RRLC 1260 equipped with a Hypercarb precolumn (3 µm bead size, 10 × 4 mm, Thermo Fisher Scientific Inc.) for inline solid-phase extraction and an ACQUITY UPLC Glycoprotein BEH Amide Column (300 Å, 1.7 µm, 2.1 mm × 50 mm, Waters Corp., Milford, MA, USA) coupled to a 6530 quadrupole time-of-flight mass spectrometer in positive mode (Agilent Technologies, Inc., Santa Clara, CA, USA). The glycans were trapped on the precolumn in 0.1% trifluoroacetic acid in 97% acetonitrile and 3% water at a flow rate of 0.25 mL/min for 4 min before the eluent was shifted to 0.1% formic acid in 97% acetonitrile for 2 min. The flow was increased to 0.625 mL/min within another 2 min and the acetonitrile concentration was subsequently increased to 40% in a linear gradient of 30 min. The fractions were collected manually, dried using a SpeedVac (Thermo Fisher Scientific Inc.) and dissolved in pure water.

The fractionated glycans were analysed using an ACQUITY UPLC I-Class PLUS equipped with a Hypercarb column (3 µm bead size, 100 × 2.1 mm, Thermo Fisher Scientific Inc.) and a SYNAPT G2-Si (Waters Corp.). Eluents A and B consisted of 10 mM NH_4_HCO_3_ and 10 mM NH_4_HCO_3_ in a mixture of 60% acetonitrile and 40% water, respectively. The elution started after 4 min at 100% eluent A, with an initial gradient to 17.2% eluent B within 1 min and then to 35% eluent B over 20 min, followed by a 5 min gradient to 100% eluent B. The mass spectrometer was set to negative mode and data-independent acquisition (MSE) was performed by applying a collision energy ramp from 30 to 55 V.

### 4.3. Analysis of Glycopeptides

Abrin (EURM-113) was precipitated with 10% (*m*/*v*) trichloroacetic acid at 4 °C, washed with ice cold acetone and dried using a SpeedVac (Thermo Fisher Scientific Inc.). The pellet was dissolved in 6 M guanidinium hydrochloride in 50 mM Tris-HCl buffer (pH 8.5) and 10 mM dithiothreitol and incubated at 56 °C for 20 min. After cooling down to room temperature, 40 mM iodoacetamide was added to alkylate free thiols at room temperature for 20 min in the dark. The unreacted iodoacetamide was quenched by the addition of 60 mM dithiothreitol. Buffer exchange to 100 mM Tris-HCl buffer, pH 8.2, containing 4 mM calcium chloride was performed using a PD SpinTrap G-25 (Cytiva) following the manufacturer’s instructions. The samples were then digested with mass-spectrometry-grade trypsin for 100 min at 37 °C or with sequencing-grade chymotrypsin 16 h at 25 °C (Promega Corp., Fitchburg, WI, USA), with an enzyme to protein ratio of 1:65 and 1:32, respectively. The enzyme reaction was quenched by adding 1% of acetic acid and diluted to 50% acetonitrile and 1% dimethyl sulfoxide. An ACQUITY UPLC I-Class PLUS equipped with ACQUITY Premier Glycan BEH Amide, 2.1 × 150 mm HILIC column (Waters Corp.) and a SYNAPT G2-Si (Waters Corp.) in positive mode was used to analyse the peptides. Separation was performed after an initial isocratic segment of 1 min with 14.2% water and 0.1% formic acid in acetonitrile by a gradient to 51% water with 0.1% formic acid in 25 min, maintaining a flow rate of 0.25 mL/min. The column was washed with a steeper gradient to 97% water and 0.1% formic acid within 0.5 min. Evaluation was performed with a BiopharmaLynx™ (Waters Corp.).

To differentiate between the glycosylation sites at N140 and N141 of the B-chain, the chymotryptic peptides were neutralised with 0.5 M Bis-Tris buffer (pH 8.0) and deglycosylated with PNGase F (Promega Corp.) for 2 h at 37 °C. The peptides were separated on an ACQUITY UPLC Peptide BEH C18, 2.1 × 50 mm column (Waters Corp.) installed in the ACQUITY UPLC I-Class PLUS with a SYNAPT G2-Si (Waters Corp.). The column temperature was set to 60 °C and the flow rate to 0.2 mL/min. Eluent A was 3% acetonitrile and 0.1% formic acid in water and eluent B was 5% water and 0.1% formic acid in acetonitrile. The gradient from 100% eluent A to 7% eluent B started after an initial isocratic segment of 1 min. The column was cleaned with a steep gradient to 100% eluent B in 5 min. A mass spectrometer was used in electron transfer dissociation (ETD) mode with 4-nitrotoluene (Sigma-Aldrich, Inc., St. Louis, MO, USA) to produce radical anions.

### 4.4. Analysis of Intact Polypeptide Chains

Abrin (EURM-113) was reduced with 25 mM tris(2-carboxyethyl)phosphine in PBS containing 25 mM lactose at 12 °C for 24 h. The analysis was performed using an ACQUITY UPLC I-Class PLUS equipped with a BioResolve RP mAb Polyphenyl Column (450Å, 2.7 µm, 2.1 mm × 150 mm, Waters Corp.) and a SYNAPT G2-Si (Waters Corp.) in positive mode. Eluent A and B consisted of 3% and 95% acetonitrile in water, respectively, with both containing 0.1% formic acid and 0.03% trifluoroacetic acid. The column temperature was set to 90 °C, the flow rate was 0.2 mL/min and the initial mobile phase was mixed with 95% eluent A and 5% eluent B. The gradient started with an increase of eluent B to 30% in 1 min, then to 46% in 20 min and finally to 100% in 2.25 min. The evaluation was performed with MassLynx and MaxEnt 1 algorithm.

## Figures and Tables

**Table 1:** Concentration of monosaccharides of hydrolysed abrin and calculated monosaccharide to protein ratio with standard deviation (*s*).

	*c* /µM	*s* /µM	Ratio/1	*s* /1
Fuc	13	2.2	0.56	0.09
Gal	13	2.1	0.55	0.09
GlcN	117	13	5.0	0.57
Glc	8	15	0.34	0.65
Xyl	25	6.7	1.1	0.29
Man	318	11	13.6	0.49

**Table 2:** Identities and shares of the released *N*-glycans of abrin. Green circle (mannose), orange star (xylose), red triangle (fucose), and blue square (*N*-acetylglucosamine). Proportions and standard deviations (SD) of the relative areas (*A*) of the peaks in the extracted ion chromatograms of the singly ([M+H]^+^) and doubly ([M+2H]^+^) charged glycans.

Label	Structure	[M+H]^+^	[M+2H]^2+^	Proportion *A*/%	SD *s*/%
M2X	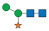	883	442	1.5	0.74
M2FX (2)	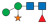	1029	515	0.6	0.14
M2FX (1)	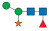	1029	515	15.3	2.52
M3X	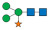	1045	523	1.0	0.04
M4 (3)	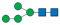	1075	538	0.5	0.16
M4 (1)	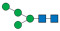	1075	538	1.1	0.75
M4X	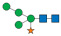	1207	604	17.2	6.18
M5 (1,3)	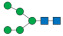	1237	619	2.0	0.09
M5 (1,2)	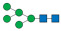	1237	619	14.5	0.35
M5X	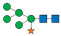	1369	685	6.8	0.17
M6	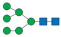	1400	700	31.8	5.52
M7 (1)	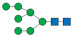	1562	781	7.3	0.48
M7 (2)	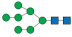	1562	781	0.4	0.11

## Data Availability

The original contributions presented in this study are included in the article/Supplementary Materials. Further inquiries can be directed to the corresponding author.

## Supplementary Materials

The following supporting information can be downloaded at https://www.mdpi.com/article/10.3390/toxins17030108/s1, Figure S1: Percent Identity Matrix of amino acid sequence alignments of abrin isoforms shows an overview of abrin sequences retrieved from Uniprot and from other sequencing data of *Abrus precatorius* genome [6]; Figure S2: Sequence alignment of abrin isoforms retrieved from Uniprot [43,44]; Table S1: Masses of abrin A- and B-chains, list of experimental masses acquired with assignment to theoretical average masses for A- and B-chains of the isoforms including *N*-glycans and other post-translational modifications [45].
