# Supplementary material for: N-Glycosylation Profile of Abrin Certified EU Reference Material
Source: Toxins (Basel). 2025 Feb 26;17(3):108. doi: 10.3390/toxins17030108 (PMC11945959; doi:10.3390/toxins17030108)
Supplement: Supplementary file 1 [file toxins-17-00108-s001.zip › toxins-3434444-supplementary-done.pdf]

toxins

MDPI

Supplementary Materials: *N*-Glycosylation Profile of Abrin Certified EU Reference Material

Roland Josuran [1], Andreas Wenger [1], Sylvia Worbs [2], Bettina Kampa [2], Andreas Rummel [3], Brigitte G. Dorner [2] and Sabina Gerber [1,*]

[1] Institute of Chemistry and Biotechnology, ZHAW Zurich University of Applied Sciences, 8820 Wädenswil, Switzerland
[2] Biological Toxins (ZBS3), Centre for Biological Threats and Special Pathogens, Robert Koch Institute, 13353 Berlin, Germany
[3] Institute of Toxicology, Hannover Medical School, 30625 Hannover, Germany
* Correspondence: sabina.gerber@zhaw.ch; Tel.: +41-58-934-54-28

1. Percent Identity Matrix of Sequence Alignments

Percent Identity Matrix

P11140_Abrin_a	100.00%	86.91%	88.45%	87.88%	87.88%	87.88%	88.07%	88.64%	76.53%	75.48%	74.47%	73.13%
Q06077_Abrin_b	86.91%	100.00%	90.32%	90.51%	90.51%	90.51%	91.46%	89.94%	72.28%	71.07%	70.77%	70.00%
Abrin_c_Protein_ID_XP_027351635_1_2018	88.45%	90.32%	100.00%	93.77%	93.77%	93.77%	94.48%	92.80%	75.14%	70.80%	71.48%	70.76%
Abrin_c_Protein_ID_XP_027349057_1_2018	87.88%	90.51%	93.77%	100.00%	100.00%	100.00%	97.69%	95.83%	76.40%	71.63%	71.12%	71.12%
Abrin_c_Protein_ID_XP_027349056_1_2018	87.88%	90.51%	93.77%	100.00%	100.00%	100.00%	97.69%	95.83%	76.40%	71.63%	71.12%	71.12%
Abrin_c_Protein_ID_XP_027349055_1_2018	87.88%	90.51%	93.77%	100.00%	100.00%	100.00%	97.69%	95.83%	76.40%	71.63%	71.12%	71.12%
P28590_Abrin_c	88.07%	91.46%	94.48%	97.69%	97.69%	97.69%	100.00%	98.30%	76.76%	71.90%	71.48%	70.94%
Q06076_Abrin_d	88.64%	89.94%	92.80%	95.83%	95.83%	95.83%	98.30%	100.00%	77.10%	72.18%	71.79%	70.44%
Abrin_d_like_Protein_ID_XP_027334134.1_2018	76.53%	72.28%	75.14%	76.40%	76.40%	76.40%	76.76%	77.10%	100.00%	80.17%	76.95%	76.95%
Abrin_a_like_Protein_ID_XP_027338619.1_2018	75.48%	71.07%	70.80%	71.63%	71.63%	71.63%	71.90%	72.18%	80.17%	100.00%	84.57%	90.66%
Abrin_a_like_Protein_ID_XP_027338618.1_2018	74.47%	70.77%	71.48%	71.12%	71.12%	71.12%	71.48%	71.79%	76.95%	84.57%	100.00%	92.06%
Abrin_a_like_Protein_ID_XP_027357184.1_2018	73.13%	70.00%	70.76%	71.12%	71.12%	71.12%	70.94%	70.44%	76.95%	90.66%	92.06%	100.00%

Figure S1: Percent Identity Matrix of amino acid sequence alignments of isoforms abrin-a (P11140), abrin-b (Q06077), abrin-c (P28590), abrin-d (Q06076) from UniprotKB with abrin-like proteins from whole genome sequence BioProject PRJNA510631 [1]. Matrix produced with https://www.uniprot.org/align.

2. Sequence Alignments of Abrin Isoforms as deposited in Uniprot

(a) A-chain

```
Abrin-a|1-251       QDRPIKFSTEGATSQSYKQFIEALRERLRGGLIHDIPVLPDPTTLQERNRYITVELSNSD      60
Abrin-b|1-250       QDQVIKFTTEGATSQSYKQFIEALRQRLTGGLIHGIPVLPDPTTLQERNRYISVELSNSD      60
Abrin-c|35-285      QDQVIKFTTEGATSQSYKQFIEALRQRLTGGLIHDIPVLPDPTTVEERNRYITVELSNSE      60
Abrin-d|1-251       QDQVIKFTTEGATSQSYKQFIEALRQRLTGGLIHDIPVLPDPTTVEERNRYITVELSNSE      60
                    **: ***:*****************:** *****.*********::******:******:

Abrin-a|1-251       TESIEVGIDVTNAYVVAYRAGTQSYFLRDAPSSASDYLFTGTDQHSLPFYGTYGDLERWA      120
Abrin-b|1-250       TESIEAGIDVSNAYVVAYRAGNRSYFLRDAPTSASRYLFTGTQQYSLRFNGSYIDLERLA      120
Abrin-c|35-285      RESIEVGIDVTNAYVVAYRAGSQSYFLRDAPASASTYLFPGTQRYSLRFDGSYGDLERWA      120
Abrin-d|1-251       RESIEVGIDVTNAYVVAYRAGSQSYFLRDAPASASTYLFPGTQRYSLRFDGSYGDLERWA      120
                     ****.****:**********.:********:*** *** **:::** * *:* **** *

Abrin-a|1-251       HQSRQQIPLGLQALTHGISFFRSGGNDNEEKARTLIVIIQMVAEAARFRYISNRVRVSIQ      180
Abrin-b|1-250       RQTRQQIPLGLQALRHAISFLQSGTD-DQEIARTLIVIIQMASEAARYRFISYRVGVSIR      179
Abrin-c|35-285      HQTREEISLGLQALTHAISFLRSGASNDEEKARTLIVIIQMASEAARYRYISNRVGVSIR      180
Abrin-d|1-251       HQTREEISLGLQALTHAISFLRSGASNDEEKARTLIVIIQMASEAARYRCISNRVGVSIR      180
                    :*:*::* ****** *.***::** . ::* **********.:****:* ** ** ***:

Abrin-a|1-251       TGTAFQPDAAMISLENNWDNLSRGVQESVQDTFPNQVTLTNIRNEPVIVDSLSHPTVAVL      240
Abrin-b|1-250       TNTAFQPDAAMISLENNWDNLSGGVQQSVQDTFPNAVTLRSVNNQPVIVDSLTHQSVAVL      239
Abrin-c|35-285      TGTAFQPDPAMLSLENNWDNLSGGVQQSVQDTFPNNVILSSINRQPVVVDSLSHPTVAVL      240
Abrin-d|1-251       TGTAFQPDPAMLSLENNWDNLSGGVQQSVQDAFPNNVILSSINRQPVVVDSLSHPTVAVL      240
                    *.****** **:********** ***:****:*** * * .:..:**:****:* :****

Abrin-a|1-251       ALMLFVCNPPN         251
Abrin-b|1-250       ALMLFVCNPPN         250
Abrin-c|35-285      ALMLFVCNPPN         251
Abrin-d|1-251       ALMLFVCNPPN         251
                    ***********
```

(b) Linker Peptide

```
Abrin-a|252-261      ANQSPLLIRS          10
Abrin-b|251-260      ANQSPLLIRS          10
Abrin-c|286-295      ANQSPLLIRS          10
Abrin-d|252-261      ANQSPLLIRS          10
                     **********
```

(c) B-chain

```
Abrin-a|262-528      IVEKSKICSSRYEPTVRIGGRDGMCVDVYDNGYHNGNRIIMWKCKDRLEENQLWTLKSDK      60
Abrin-b|261-527      IVEKSKICSSRYEPTVRIGGRNGMCVDVYDDGYHNGNRIIAWKCKDRLEENQLWTLKSDK      60
Abrin-c|296-562      IVEESKICSSRYEPTVRIGGRDGMCVDVYDDGYHNGNRIIAWKCKDRLEENQLWTLKSDK      60
Abrin-d|262-528      IVEESKICSSRYEPTVRIGGRDGMCVDVYDDGYHNGNRIIAWKCKDRLEENQLWTLKSDL      60
                     ***:*****************:********:********* ******************

Abrin-a|262-528      TIRSNGKCLTTYGYAPGSYVMIYDCTSAVAEATYWEIWDNGTIINPKSALVLSAESSSMG      120
Abrin-b|261-527      TIRSNGKCLTTEGYAPGNYVMIYDCTSAVAEATYWEIWDNGTIINPKSALVLSAESSSMG      120
Abrin-c|296-562      TIRSNGKCLTTEGYAPGNYVMIYDCTSAVAEATYWEIWDNGTIINPKSALVLSAESSSMG      120
Abrin-d|262-528      TIRSNGKCLTTEGYAPGNYVMIYDCTSAVAEATYWEIWDNGTIINPKSALVLSAESSSMG      120
                     *********** *****.******************************************

Abrin-a|262-528      GTLTVQTNEYLMRQGWRTGNNTSPFVTSISGYSDLCMQAQGSNVWMADCDSNKKEQQWAL      180
Abrin-b|261-527      GTLTVQTNEYLMRQGWRTGNNTSPFVTSISGYSDLCMQAQGSNVWLAYCDNNKKEQQWAL      180
```

```
Abrin-c|296-562      GTLTVQTNEYLMRQGWRTGNNTSPFVTSISGYSDLCMQAQGSNVWLADCDNNKKEQQWAL      180
Abrin-d|262-528      GTLTVQTNEYLMRQGWRTGNNTSPFVTSISGYSDLCMQAQGSNVWLADCDNNKKEQQWAL      180
                     *********************************************:* **.*********

Abrin-a|262-528      YTDGSIRSVQNTNNCLTSKDHKQGSTILLMGCSNGWASQRWVFKNDGSIYSLYDDMVMDV      240
Abrin-b|261-527      YTDGSIRSVQNTNNCLTSKDHKQGSPIVLMACSNGWASQRWLFRNDGSIYNLHDDMVMDV      240
Abrin-c|296-562      YTDGSIRSVQNTNNCLTSKDHKQGSPIVLMACSNGWASQRWLFKNDGSIYNLHDDMVMDV      240
Abrin-d|262-528      YTDGSIRSVQNTNNCLTSKDHKQGSPIVLMACSNGWASQRWLFKNDGSIYSLYDDMVMDV      240
                     ************************* *:**.**********:*:******.*:*******

Abrin-a|262-528      KGSDPSLKQIILWPYTGKPNQIWLTLF      267
Abrin-b|261-527      KRSDPSLKEIILHPYHGKPNQIWLTLF      267
Abrin-c|296-562      KRSDPSLKEIILHPYHGKPNQIWLTLF      267
Abrin-d|262-528      KGSDPSLKQIILWPYTGKPNQIWLTLF      267
                     * ******:*** ** ***********
```

Figure S2: Sequence alignment [2] of abrin isoforms. Sequences were taken from Uniprot [3]. UniprotKB accession numbers: Abrin-a P11140, abrin-b Q06077, abrin-c P28590, abrin-d Q06076. *N*-glycosylation motifs are bold and underlined, glycosylated asparagine residues are highlighted in green, glycopeptides are labelled in yellow and peptides covering the *N*-glycosylation motif at N200 are highlighted in cyan, tryptic peptides in bold, italics and with dotted lines are unique for abrin-d among the four isoforms.

3. Mass Table

Table S1. Masses of abrin A- and B-chains. Average molecule masses were calculated considering modifications using Prot pi [4]. N-terminal pyroglutamate (pE), additional C-terminal amino acids (+AN) and *N*-glycosylation were confirmed by peptide analysis. n.d. not detectable.

Molecule	Modification	Calculated Mass	Experimental Mass
Abrin-a, A-chain		28'097 Da	28’096 Da
Abrin-a, A-chain	pE	28'080 Da	28’081 Da
Abrin-b, A-chain	pE, 2•M2FX	29'853 Da	29’855 Da
Abrin-b, A-chain	pE, +AN, 1•M2FX	29'029 Da	29’031 Da
Abrin-b, A-chain	pE, +AN, 2•M2FX	30'038 Da	30’040 Da
Abrin-c, A-chain	pE	27'858 Da	27’860 Da
Abrin-d, A-chain	pE	27'768 Da	n.d.
Abrin-a, B-chain	M6, M4X[1]	32'650 Da	32’646 Da
Abrin-b, B-chain	M6, M4X[1]	32'766 Da	n.d.
Abrin-c, B-chain	M6, M4X[1]	32'692 Da	n.d.
Abrin-d, B-chain	M6, M4X[1]	32'589 Da	n.d.

[1] Most common combination of B-chain glycosylation.

References

1. Hovde B. T.; Daligault H. E.; Hanschen E. R.; Kunde Y. A.; Johnson M. B.; Starkenburg S. R.; Johnson S. L. Detection of Abrin-Like and Prepropulchellin-Like Toxin Genes and Transcripts Using Whole Genome Sequencing and Full-Length Transcript Sequencing of Abrus Precatorius. *Toxins* **2019**, *11*, doi:10.3390/toxins11120691.
2. Madeira, F.; Madhusoodanan, N.; Lee, J.; Eusebi, A.; Niewielska, A.; Tivey, A.R.N.; Lopez, R.; Butcher, S. The EMBL-EBI Job Dispatcher Sequence Analysis Tools Framework in 2024. *Nucleic Acids Res* **2024**, *52*, W521–W525, doi:10.1093/nar/gkae241.
3. The UniProt Consortium UniProt: The Universal Protein Knowledgebase in 2025. *Nucleic Acids Research* **2024**, gkae1010, doi:10.1093/nar/gkae1010.
4. Protein Tool Available online: https://www.protpi.ch/Calculator/ProteinTool (accessed on 11 December 2024).
